# Protocol: Optimised methodology for isolation of nuclei from leaves of species in the *Solanaceae* and *Rosaceae* families

**DOI:** 10.1186/1746-4811-9-31

**Published:** 2013-07-26

**Authors:** Sidona Sikorskaite, Minna-Liisa Rajamäki, Danas Baniulis, Vidmantas Stanys, Jari PT Valkonen

**Affiliations:** 1 Institute of Horticulture, Lithuanian Research Centre for Agriculture and Forestry, Kaunas st 30, Babtai, LT-54333, Kaunas, Lithuania; 2 Department of Agricultural Sciences, University of Helsinki, PO Box 27, FIN-00014 Helsinki, Finland

**Keywords:** Nuclei isolation, Solanaceae, Rosaceae, Tobacco, Potato, Apple

## Abstract

In this study, a protocol is described for rapid preparation of an enriched, reasonably pure fraction of nuclear proteins from the leaves of tobacco (*Nicotiana tabacum*), potato (*Solanum tuberosum*) and apple (*Malus domestica*). The protocol gives reproducible results and can be carried out quickly in 2 hours. Tissue extracts clarified with filtration were treated with non-ionic detergent (Triton X-100) to lyse membranes of contaminating organelles. Nuclei were collected from a 60% Percoll layer of density gradient following low-speed centrifugation. Western blot analysis using antibodies to marker proteins of organelles indicated that the nuclear protein fractions were highly enriched and free or nearly free of proteins from the endoplasmic reticulum and chloroplasts.

## Background

The nucleus is a highly specialized, complex and heterogeneous organelle of the cell. It contains most of the cell’s hereditary information and controls most of the cellular functions [1,2]. The studies on transcription of genes [3], synthesis and processing of RNA [4], and posttranscriptional gene silencing [5] are enhanced by the availability of isolated nuclei. Isolated nuclei are also used to obtain high-quality chromosomal DNA with minimized chloroplast and mitochondrial DNA contamination [6], to determine ploidy level, and to measure nuclear DNA content using flow cytometry [7-11].

Nuclear proteins play a central role in regulating gene expression. Studies on nuclear proteome have been conducted in model species, such as *Arabidopsis thaliana*[12,13], *Oryza sativa*[14], and *Medicago truncatula*[15], as well as in chickpea (*Cicer arietinum*) [16]. The studies have estimated the size of the proteome, placed the identified proteins by functional classes, revealed targeting of the proteins and their posttranslational modifications. Pendle *et al*. [17] conducted the first proteomic analysis of nucleoli of *A*. *thaliana*, whereas Calikowski *et al*. [18] characterised nuclear matrix in *Arabidopsis*. Tan *et al*. [19] performed proteomic and phosphoproteomic analysis of the chromatin-associated proteins in rice, whereas Aki *et al*. [20] identified nuclear proteins involved in evolutionarily conserved mechanisms for sugar response in plants. Nuclear proteome analyses have also revealed major changes in nuclear protein composition during the influence of environmental stimuli [12,21,22]. Species-specific or tissue-specific comparative analyses of nuclear proteomes have provided new insights into understanding of the composition of the nucleus. Repetto *et al*. [15] compared nuclear proteomes of seeds of *Medicago*, leaves of *Arabidopsis* and seedlings of chickpea. On the other hand, Choudhary *et al*. [22] performed a comparative analysis of nuclear proteome at the organism level and assessed differences in protein composition of the samples isolated from rice, *Arabidopsis*, *Medicago* and chickpea.

Isolation of nuclei from protoplasts is commonly used and provides high yields of pure nuclei [23,24]. However, the protocols for protoplast preparation are laborious and available for a limited number of species. Therefore, methods for isolation of nuclei from intact plant cells and tissues have been developed for a number of plant species using embryos [25,26], seed coat [27], cultured plant cells [28], meristematic root tissues [29] or leaf tissues [30,31]. Most of the isolation methods use the same steps of the procedure, including tissue disruption, filtration, centrifugation, solubilisation of membranes of contaminating organelles with non-ionic detergents, and separation of nuclei by density gradient centrifugation. Preparation of samples for flow cytometry usually requires only the first three steps [7]. Non-ionic detergents, such as Triton X-100, facilitate the release of nuclei from cells and prevent nuclei from clumping [32]. Moreover, application of detergent is crucial for isolation of nuclei from green tissue, in which nuclei need to be separated from chloroplasts by disrupting the latter with the detergent [33]. However, high concentrations of the detergent or prolonged exposure to it may also disrupt the nuclear membrane [34,35]. To facilitate analysis of nuclei from different types of cells, Deal and Henikoff [36] developed a method for isolation of nuclei tagged in specific cell types (INTACT), which allows isolation of biotin-labelled nuclei from specific types of cells of a tissue by streptavidin-coated magnetic beads. The method provides high purity of nuclei, but requires construction of transgenic plants for tissue-specific expression of affinity-labelled nuclear envelope protein, which is time consuming and not easily applicable for many plant species.

Different buffers for isolation of nuclei have been reported with variable compositions according to the plant material and the purpose of study for which the nuclei are isolated. During the release of nuclei from intact cells, nuclear isolation buffers must ensure the integrity of nuclear membrane and stability of nuclei throughout the experiment. Organic buffers (MES, HEPES, PIPES, TRIS, MOPS) are required to stabilize pH in the solution. Inorganic salts (KCl, NaCl) maintain ionic strength of the solution, and certain organic agents (sucrose, glycerol, hexylene glycol) stabilize membranes [11]. Polyamines in the presence of metal chelators or divalent cations help to stabilize the nuclear chromatin and to avoid aggregation of nuclei [28]. Reducing compounds, such as β-mercaptoethanol and dithiothreitol (DTT), are crucial for nuclear protein preparations, because they maintain cysteine residues in reduced form. The requirement for protease inhibitors depends on further application of the isolated nuclei. While protease inhibitors are used in some studies [34,37], they are omitted from the buffers used for intact nuclei in other investigations [3,12]. Many plant species contain large amounts of phenolic compounds and the use of reducing agents and resins absorbing phenolics (such as polyvinylpyrrolidone) is needed [10,16].

Plant species and tissues vary in their physico-chemical composition. Therefore, adjustment and optimization of the protocols are needed to obtain pure preparations of intact nuclei that are free from contaminating non-nuclear proteins. The aim of this study was to develop a simple protocol applicable to isolation of nuclei and nuclear proteins from leaf tissues of three different plant species representing the families *Solanaceae* and *Rosaceae*. This was achieved by modification of the protocol of Cushman [31] by adjusting the concentration of detergents used for lysis of organelles and the parameters of density gradient and centrifugation, which were dependent on species-specific size and density of the nuclei. Furthermore, adjustments were made to remove phenolics and to stabilize protein samples for proteomics analyses. The nuclei-enriched fractions obtained with the optimised protocol showed negligible contamination with plastids, other cellular organelles and non-nuclear proteins. The isolation procedure could be concluded in 2 h.

## Materials and methods

### Reagents

2-(4-amidinophenyl)-1H-indole-6-carboxamidine (DAPI) (Carl-Roth cat. No. 6335.1)

2-(*N*-morpholino)ethanesulfonic acid (MES) (Carl-Roth cat. No. 4256.2)

3-[(3-cholamidopropyl)dimethylammonio]-1-propanesulfonate (CHAPS) (Carl-Roth cat. No. 1479.2)

4-(2-hydroxyethyl)-1-piperazineethanesulfonic acid (HEPES) (Carl-Roth cat. No. 9105.4)

Bradford Reagent (Sigma cat. No. B6916)

Diethyl ether (Carl-Roth cat. No. 5920.2)

Dithiothreitol (DTT) (Carl-Roth cat. No. 6908.2)

Ethylenediaminetetraacetic acid (EDTA) (Sigma cat. No. EDS)

Glycerol (Sigma cat. No. 49781)

Liquid nitrogen

MgCl_2_ hexahydrate (Carl-Roth cat. No. HN03.2)

Percoll (Sigma cat. No. P1644)

Polyvinylpyrrolidone (PVP) (Sigma cat. No. PVP40)

Potassium chloride (KCl) (Fluka cat. No. 60128)

Potassium phosphate monobasic (KH_2_PO_4_) (Sigma cat. No. P8416)

Protease inhibitor cocktail (Sigma cat. No. P9599)

Sodium chloride (NaCl) (Carl-Roth cat. No. 9265.1)

Sodium dodecyl sulfate (SDS) (Carl-Roth cat. No. 2326.2)

Sodium phosphate dibasic (Na_2_HPO_4_) (Sigma cat. No. S9763)

Spermidine (Sigma cat. No. S0266)

Spermine (Carl-Roth cat. No. 7162.1)

Sucrose (Sigma cat. No. S0389)

Thiourea (Sigma cat. No. T7875)

Triton X-100 (Carl-Roth cat. No. 3051.2)

TRizol reagent (Invitrogen cat. No. 15596–026)

Urea (Bio-Rad cat. No. 161–0730)

ECL anti-rabbit IgG, Horseradish Peroxidase-linked whole antibody (GE Healthcare cat. No. NA934V)

Rabbit anti-histone H3 (Agrisera cat. No. AS10 710)

Rabbit anti-lumenal-binding protein 2 (Agrisera cat. No. AS09 481)

Rabbit anti-plastocyanin (Agrisera cat. No. AS06 141)

SuperSignal West Pico Chemiluminescent Substrate (Thermo Scientific cat. No. 34080)

### Equipment

Blades

Cheesecloth

Falcon tubes (15 ml, 50 ml)

Funnels

Glass container

Homogenizer (UltraTurrax T25, IKA)

Hybond-P polyvinylidene fluoride membrane (GE Healthcare)

Leitz Laborlux S microscope with an epifluorescence extension (Leitz Ploemopak) and DAPI filter

Miracloth

Mortar and pestle

Orbital shaker incubator

Pasteur pipettes

Swinging rotor centrifuge (*e*.*g*., Eppendorf 5804R)

### Solutions

Nuclei isolation buffer (NIB)

1× NIB: 10 mM MES-KOH (pH 5.4), 10 mM NaCl, 10 mM KCl, 2.5 mM EDTA, 250 mM sucrose, 0.1 mM spermine, 0.5 mM spermidine, 1 mM DTT.

4× stock solution of NIB was prepared without spermine, spermidine and DTT, and stored at 4°C. 1× NIB was prepared from the 4× stock and supplemented immediately before use with spermine, spermidine and DTT from the stocks of 100 mM spermine, 100 mM spermidine and 1 M DTT in deionized H_2_O.

1× NIB was used for nuclei isolation from tobacco and potato leaves, whereas for apple leaf tissue, 1× NIB was further supplemented with 1% PVP (MW 40,000) and 0.1% protease inhibitor cocktail.

**10% (v/v) Triton X-100 in deionized H**_**2**_**O**

60% (v/v) Percoll solution in 1×NIB

2.5 M sucrose

**Nuclei storage buffer**: 20% glycerol, 20 mM HEPES-KOH (pH 7.2), 5 mM MgCl_2_, 1 mM DTT. Store at −20°C.

**1× phosphate-buffered saline (PBS)**: 137 mM NaCl, 2.7 mM KCl, 10 mM Na_2_HPO_4_, 1.8 mM KH_2_PO_4_, pH 7.4.

**Electrophoresis buffer**: 8.75 M urea, 2.5 M thiourea, 5% (w/v) CHAPS.

### Plant material

Tobacco plants (*Nicotiana tabacum* L. cultivar Samsun nn) were grown from seeds. Potato plants (*Solanum tuberosum* L. breeding line v2-108 [38]) were propagated in tissue culture, plantlets transferred to soil in pots and multiplied by rooting stem cuttings. Plants were grown under controlled conditions in a growth chamber at 23°C with photoperiod of 16 h (110 μE m^-2^ s^-1^) under illumination of fluorescent lamps (tubes of 58 W/830 and 36 W/77 in turns) and relative humidity of 40%. Plants were watered twice a week and fertilized weekly with 1% N:P:K = 16:9:22 fertilizer. The fresh leaves of tobacco (BBCH growth stage code: 1004 [39]; leaf length of 8–11 cm) and potato (BBCH growth stage code: 19 [39]; leaflet lengths of 2–5 cm) plants were sampled in the morning (2–4 hours to the light period) for isolation of nuclei. The leaves of young apple (*Malus domestica* Borkh. cultivar Orlovim) (BBCH growth stage code: 19 [39]; leaf length of 5–7 cm) were collected in June (before noon) from the young shoots of field grown trees in the genetic resources collection of the Institute of Horticulture, Lithuania and stored at −70°C after quick freezing in liquid nitrogen.

### Protocol

**Figure 1:**
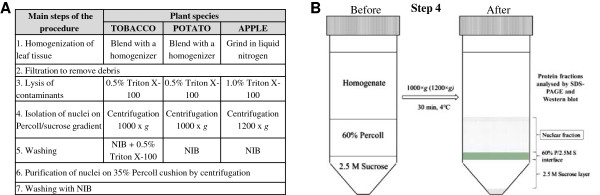
**Outline of the procedures used for isolation of nuclei. A)** Species-specific adjustments at specific steps of isolation of nuclei from the leaves of tobacco, potato and apple. NIB, buffer for isolation of nuclei. **B)** Isolation of nuclei using Percoll/sucrose density gradient centrifugation (step 4) shown in more detail. The crude preparation of nuclei is loaded on the top of 60% Percoll/2.5 M sucrose density gradient before centrifugation. After centrifugation (1000 × *g* for tobacco and potato, 1200 × *g* for apple), the Percoll layer contains most of the nuclei (see Nuclear fraction) and is collected for further purification on 35% Percoll cushion. Also the two other fractions (60% P/2.5 M S interface and 2.5 M Sucrose layer) were subjected to western blot analysis (see Figure 2). The shaded fraction on the bottom of the tube after centrifugation contains mainly starch grains.

An overview of the main steps of the protocol is presented in Figure 1.

### Isolation of nuclei

NOTE: All solutions, glassware, tubes, and equipment should be precooled to 0-4°C and kept on ice all the time. Homogenizer heads and centrifuge rotors should be cooled down to the same temperature.

#### Homogenization of leaf tissue

***Solanaceae*** Remove the midvein from leaves and chop 3–5 g of them into small pieces with a blade. Treat with ice-cold diethyl ether (2–3 ml/g) for 3–5 min in a glass container. Rinse ether-drained leaves with ice-cold NIB (3–5 ml/g fresh weight) and discard rinse. Grind in 5–10 volumes of ice-cold NIB in 50 ml Falcon tube with homogenizer set on its lowest speed (8000 rpm) 3–5 times, each 5 s, until the tissue is completely disrupted.

NOTE: According to our observations, nuclei of potato are more fragile than those of tobacco and should be handled with special care.

##### *Rosaceae*

Grind frozen apple leaves (2 g) in liquid nitrogen into powder with mortar and pestle and resuspend in ten volumes of NIB + 1% PVP (approximately 20 ml).

NOTE: diethyl ether treatment was not used for homogenization of apple leaves.

#### Filtration

***Solanaceae*** Slowly decant homogenates through two layers of pre-wetted cheesecloth and then through one layer of pre-wetted Miracloth.

##### *Rosaceae*

Slowly decant homogenates through two layers of pre-wetted cheesecloth. Remove the debris from cheesecloth, resuspend in another 20 ml of NIB + 1% PVP and pass through the same cheesecloth. Filter through one layer of pre-wetted Miracloth.

NOTE: It is important to use as small a pad of cheesecloth and Miracloth as possible to reduce the loss of sample.

#### Lysis of contaminating organelles

Add 10% Triton X-100 dropwise to the solution until a final concentration of 0.5% (tobacco and potato) or 1% (apple) is reached. Gently agitate the solution for 20 min at +4°C.

#### Centrifugation

Centrifuge the homogenate at 1000 × *g* (tobacco and potato) or 1800 × *g* (apple) for 10 min. Slowly resuspend the pellet with Pasteur pipette in 10 ml (tobacco and potato) or 5 ml (apple) of NIB.

#### Assembly of density gradient

***Solanaceae*** Place 5 ml of 2.5 M sucrose into the chilled 50 ml Falcon tube. Carefully overlay with Pasteur pipette 5 ml of 60% Percoll solution. Be very careful not to mix the sucrose and Percoll layers.

##### *Rosaceae*

Carefully with Pasteur pipette overlay 3 ml of 60% Percoll solution on 3 ml of 2.5 M sucrose in a chilled 15 ml Falcon tube.

#### Isolation of nuclei using Percoll/sucrose density gradient centrifugation

Carefully load the crude preparation of nuclei on the top of the density gradient by drawing the extract into a Pasteur pipette and slowly releasing the solution onto the side of the tube above the 60% Percoll layer (Figure 1B).

Subject the gradient to centrifugation in a swinging bucket rotor at 1000 × *g* (tobacco and potato) or 1200 × *g* (apple) for 30 min at 4°C. Use a slow break speed.

Remove the liquid above the gradient. Collect the 60% Percoll layer that contains most of the nuclei carefully with a Pasteur pipette (Figure 1B). Be careful not to disturb the dark green band located at the interface between Percoll and sucrose layers, which contains most of the contaminating debris and organelles.

#### Washing

***Solanaceae*** Dilute the Percoll suspension containing tobacco nuclei slowly with 5 volumes of NIB and 0.5% Triton X-100 using a Pasteur pipette and incubate for 10 min under gentle shaking. Dilute the Percoll suspension containing potato nuclei with 5 volumes of NIB. Centrifuge at 1000 × *g* for 10 min.

##### *Rosaceae*

Dilute the Percoll suspension with 3–5 volumes of NIB and centrifuge at 1800 × *g* for 10 min.

#### Purification of nuclei on 35% Percoll cushion

Resuspend the pellet of nuclei in 5 ml of NIB and overlay the solution on 5 ml (tobacco and potato) or 3 ml (apple) of 35% Percoll solution. Centrifuge at 1000 × *g* (tobacco and potato) or 1200 × *g* (apple) for 10 min.

Wash the nuclei by resuspending the pellet in 5 ml of NIB and centrifugate as previously (washing step). Proceed to nuclear protein isolation or alternatively, resuspend the nuclei in 500 μl of nuclei storage buffer, freeze in liquid N_2_ and store at −70°C until use.

### Protein extraction and quantification

Total proteins are extracted using TRizol reagent according to the manufacturer’s instructions (Invitrogen). Protein concentration can be determined using Bradford reagent.

### Western blot analysis

Proteins solubilized in electrophoresis buffer are analyzed on a 12.5% SDS polyacrylamide gel by electrophoresis and electroblotted onto a Hybond-P polyvinylidene fluoride membrane. To test the purity of nuclear proteins, membranes can be probed with rabbit anti-histone (H3, 1:15.000 dilution), anti-lumenal-binding protein 2 (BiP2, 1:2.000 dilution), and anti-plastocyanin (PC, 1:6.000 dilution) polyclonal antibodies. Signals are detected by incubation with donkey anti-rabbit IgG Horseradish Peroxidase –linked whole antibody (1:20.000 dilution) using the enhanced chemiluminescence (ECL) system and SuperSignal West Pico Chemiluminescent Substrate.

### DAPI staining and microscoping

Nuclei were stained with DAPI (1 μg/ml in 1× PBS, 10 μl of nuclei in storage solution was mixed in 10 μl of DAPI solution) and analysed using a Leitz Laborlux S microscope with an epifluorescence extension and DAPI filter.

## Comments

Subcellular organelles have similar physical properties and are distributed similarly in the conventional density gradients following centrifugation, which hampers preparation of pure nuclear fractions. The protocol for isolation of nuclei should be as simple as possible, easy to repeat and yield intact and pure nuclei. Initially, we tested many published protocols for isolation of nuclei from leaves of tobacco but found the results unsatisfactory. The method of Guilfoyle [33] resulted in a good yield of intact nuclei, but with a high chloroplast contamination. When purification of nuclei on density gradient was excluded, as in Zhang *et al*. [30], very low yields of intact nuclei were obtained from leaves of apple. Direct extraction of nuclear proteins, as described by Busk and Pagés [40], yielded low amounts of nuclear proteins and the time required for preparation of buffers and the whole isolation procedure was excessive. Different density gradients (Percoll/sucrose gradient, discontinuous sucrose or Percoll gradients) were tested. Sedimentation of the nuclei at the interface with other, contaminating organelles was experienced with all methods and media used in the two-step gradients. Ultimately, we found that centrifugation on a Percoll/sucrose density gradient followed by another low-speed centrifugation was efficient for isolation of considerably pure nuclei from the three fairly different plant species. In the presented method, the difference to the method described by Cushman [31] is that β-mercaptoethanol is replaced with less pungent and toxic DTT. The buffer for isolation of nuclei is supplemented with PVP to remove phenolic compounds from samples of apple (woody plant species). The concentration of detergent (Triton X-100) was optimised for each plant species. The protocol utilizes a Percoll/sucrose density gradient and low speed centrifugation to separate nuclei from cellular debris and organelles.

### Choice of buffer for isolation of nuclei

Cushman’s [31] buffer for isolation of nuclei contains polyamines in conjunction with EDTA, which help to stabilize chromatin and nuclear proteins. Mg^2+^ used as a chromatin stabilizer in some other protocols caused severe aggregation of nuclei in our study (as observed with a microscope), presumably due to the precipitates of released chromatin interacting with Mg^2+^[34]. Buffers with pH 7.2-7.8 have been used for isolation of nuclei from leaf tissues, however, Cushman’s [31] MES-KOH buffer with a relatively low pH (pH 5.8) used in our experiments had no adverse effects. Moreover, buffers with a low pH have been reported to improve nuclear yield with minimal cytoplasmic contamination and clumping of nuclei from plant protoplasts [24] and produce more stable nuclei from animal cells [28].

### Leaf homogenization and filtration

A major obstacle to successful tissue fractionation is experienced with disruption of plant cell wall, because any treatment that ruptures the cell can also disrupt the nucleus. Blender-type homogenizers give better results with many types of plant tissues, as compared with homogenization in liquid nitrogen with a mortar and a pestle [41]. On the other hand, Zhang *et al*. [30] compared the two methods (blender vs. mortar and pestle) for isolation of nuclei from tomato leaves (*Solanum lycopersicum* L.) and observed 57% and 95% of the nuclei remaining intact, respectively. We used an IKA UltraTurrax T25 homogenizer at the lowest speed possible (8000 rpm) to disrupt cells of tobacco and potato leaves after pre-treatment of tissue with diethyl ether, which facilitates cell disruption by removing cuticular waxes [42]. Apple leaf tissue was stored at −70°C and ground in liquid nitrogen to a fine powder for isolation of nuclei. According to Guilfoyle [33], freezing of plant tissues may lead to disintegration of nuclei and thus fresh leaf material should be preferred, but our observations did not indicate any significant problems with the use of frozen leaf material. Similarly, Zhang *et al*. [30] observed that grinding tomato leaves in liquid nitrogen resulted in higher proportions of intact nuclei than preparation from fresh leaves treated with a kitchen blender.

### Cross-contamination with other subcellular structures

#### Detergent

Adjustment of detergent concentration requires tissue-specific and species-specific optimisation to avoid damage to nuclear membrane. The loss of outer nuclear membrane makes nuclei more fragile and causes DNA leakage, which consequently results in aggregation of nuclei [43]. Our experiments using different Triton X-100 concentrations (0.1-1.2%) showed that an optimal concentration for lysis of contaminating organelles was 1% for apple and 0.5% for tobacco and potato. Possibly, the higher tolerance of detergent exhibited by the nuclei of apple might be associated with a smaller size as compared with the nuclei of solanaceous species (see Comment section “Integrity of nuclei”).

#### Purification of nuclei by density gradient centrifugation

Subcellular fractionation by centrifugation is based on differences in physical properties including the size (shape), buoyant density and surface charge density of organelles [44]. In a tissue homogenate, the nuclei are considered as the largest organelles. However, a nuclear pellet produced by low-speed centrifugation of a homogenate for 10 min at 500–1000 × *g* contains membrane sheets, starch grains and unbroken cells [44]. Thus, continuous, self-generating and discontinuous Percoll gradients or sucrose gradients are used to purify a “low-speed” or crude nuclear pellet. Sucrose density gradients are routinely used for organelle separation, but their disadvantage compared with Percoll gradient is that organelles might burst due to the osmotic shock caused by high sucrose concentrations [45]. Therefore, a two-step gradient is used that includes a layer of Percoll over sucrose.

We developed an alternative approach to isolate nuclei from Percoll layer, in contrast to the conventionally used gradients in which nuclei are collected from the interface of sucrose and Percoll layers. Our observations indicated that chloroplasts and starch grains concentrated with nuclei in the same region of the conventionally used Percoll/sucrose gradient. Therefore, we increased the Percoll concentration to 60% to keep the nuclei floating on top of the gradient during low-speed centrifugation, whereas most of the contaminating material and unbroken cells concentrated to the interface between Percoll and sucrose layers or penetrated deeper into the gradient (Figure 1B). During subsequent purification of nuclei through 35% Percoll, low density contaminating particles are separated with Percoll and nuclei are collected in the pellet.

### Quality analysis on isolated nuclei

**Figure 2:**
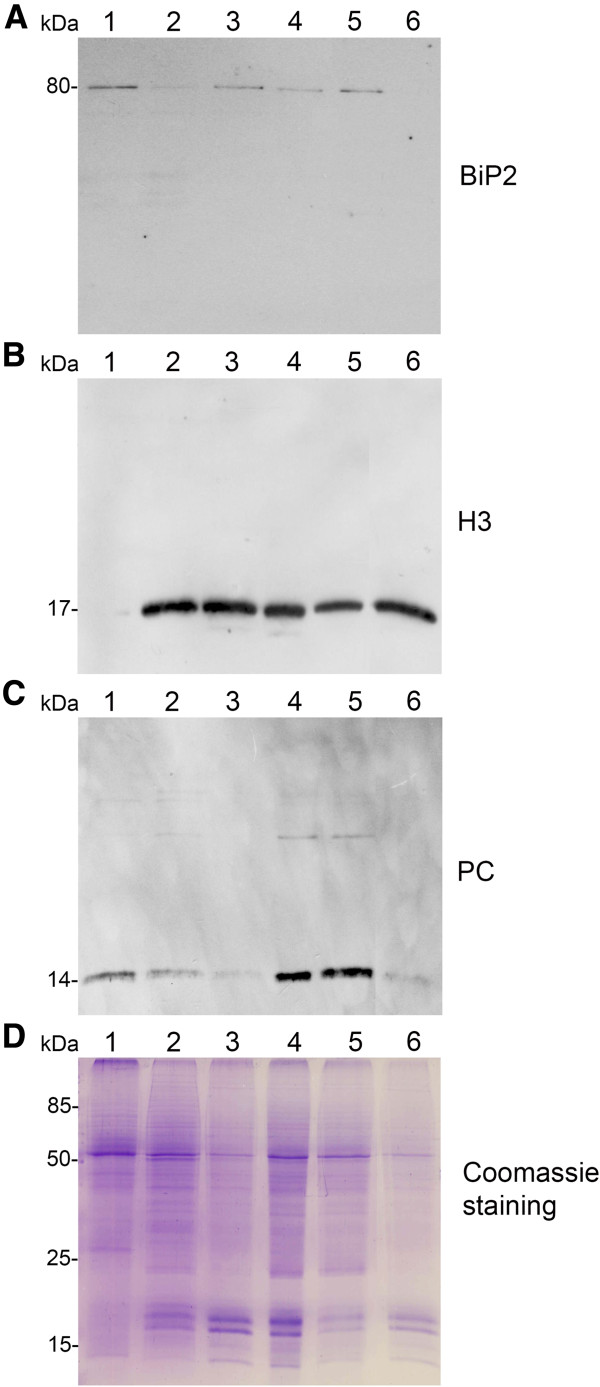
**Analysis of protein fractions from different steps of nuclei isolation.** The protein fractions obtained following the main steps of the procedure used for isolation of nuclei from leaves of apple are shown. Equal amounts of proteins (4 μg) from each fraction were loaded on the gel. **A)** Lumenal-binding protein 2 (BiP2), **B)** histone H3 and **C)** plastocyanin (PC) were detected with specific antibodies by western blot analysis. **D)** Coomassie blue staining of the fractionated proteins separated by polyacrylamide gel electrophoresis. Lane 1: crude extract of proteins from homogenized leaf tissue; lane 2: resuspended pellet of the whole cell lysate obtained following treatment with Triton X-100 and centrifugation; lane 3: nuclei collected from the 60% Percoll layer (see nuclear fraction in Figure 1B); lane 4: the interface fraction of 60% Percoll and 2.5 M sucrose layers in the density gradient containing chloroplasts and unbroken cells (see 60% P/2.5 M S interface in Figure 1B); lane 5: sucrose layer of the density gradient (see 2.5 M sucrose layer in Figure 1B); lane 6: nuclei purified by centrifugation on a 35% Percoll cushion.

Purity of the isolated nuclear fractions was evaluated by western blot analysis. Enrichment of nuclear fractions and contamination with non-nuclear proteins were analysed using specific antibodies for the nuclear protein histone H3, endoplasmic reticulum protein BiP2, and the chloroplast protein plastocyanin (PC), as exemplified with nuclear fractions of apple leaves in Figure 2. Results showed that the fraction obtained from the 60% Percoll layer of the density gradient (Figure 2, lane 3) was highly enriched with H3, as compared with the crude extract of proteins isolated from the leaf tissue (lane 1). The final nuclear protein fraction (lane 6), obtained after purification on 35% Percoll layer, was relatively pure, as indicated by absence of detectable amounts of BiP2 and the significantly reduced amounts of PC. H3 detected in the 60% Percoll/sucrose interface (lane 4) and in the sucrose layer (lane 5) corresponds to nuclear proteins sedimented with cell fragments and unbroken cells. Similar results were obtained with the nuclear protein fractions of tobacco and potato, indicating that the method works well for enrichment of nuclear fractions and results in only low contamination of other proteins. Amounts of nuclear proteins obtained with the method were 20–28 μg per g of tobacco and potato leaves and 10–17 μg per g of leaves of apple.

#### Integrity of nuclei

**Figure 3:**
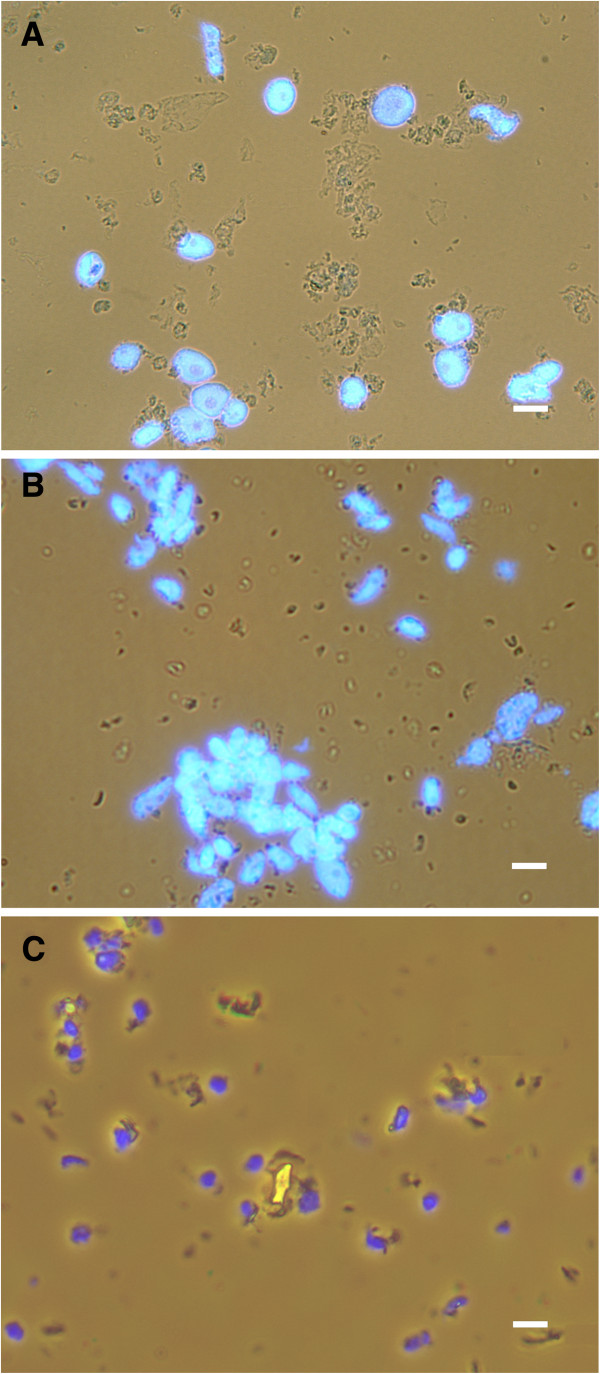
**Preparations of nuclei obtained following the final purification on 35% Percoll cushion by centrifugation.** Preparations of nuclei from leaves of **(A)** tobacco, **(B)** potato and **(C)** apple obtained after the final purification on 35% Percoll cushion by centrifugation (step 6 in Figure 1A) contained only negligible amounts of debris. DAPI-stained nuclei (shown in blue) were observed with a fluorescence microscope using UV-light and debris observed simultaneously under normal light. The unstained structures are remaining contaminants. Scale bars, 10 μm.

Integrity of the isolated nuclei and chromatin was analysed by staining with DAPI and observation with a light and fluorescence microscope. The nuclei of tobacco (Figure 3A) and potato (Figure 3B) were uniform, approximately 12 μm and 7 μm in diameter, respectively, and contained a readily visible nucleolus. The nuclei of apple (Figure 3C) were not uniform in shape and had an average diameter of 5.5 μm.

## Conclusion

The nuclear proteome has recently gained importance in basic and applied research, because identification of novel nuclear proteins helps to understand protein functions and plant responses to different environmental cues. Comparative analysis of nuclear proteomes of different plant species may also provide evolutionary insights. The protocol described here allows enrichment of the nuclear fraction and reduction of contaminating non-nuclear proteins. Collection of nuclei from a 60% Percoll layer of the density gradient following low-speed centrifugation enhances the procedure significantly and allows completion of the whole process in 2 hours. Western blot analysis of organelle-specific marker proteins and microscopic observations indicated that the fractions of nuclear proteins were highly enriched and contained no or barely detectable amounts of proteins from chloroplasts and the endoplasmic reticulum. It is suggested that the procedure described in this study may be widely applicable for isolation of nuclei from leaves of species in the families of *Solanaceae* and *Rosaceae*.

## Competing interests

The authors declare that they have no competing interests.

## Authors’ contributions

SS carried out most of the experiments. MLR participated in the experiments on extraction of nuclei from potato leaves. SS, MLR, DB, VS and JPTV planned experiments. SS, MLR, DB and JPTV interpreted results and wrote the manuscript. All authors read and approved the final manuscript.

## References

[B1] Shaw PJ Nuclear organization in plants Essays in Biochem 1996 31 77 89 9078459

[B2] Shaw PJ Brown JW Plant nuclear bodies Curr Opin Plant Biol 2004 7 6 614 620 10.1016/j.pbi.2004.09.011 15491908

[B3] Folta KM Kaufman LS Isolation of *Arabidopsis* nuclei and measurement of gene transcription rates using nuclear run-on assays Nat Protoc 2006 1 6 3094 3100 10.1038/nprot.2006.471 17406505

[B4] Park MY Wu G Gonzalez-Sulser A Vaucheret H Poethig RS Nuclear processing and export of microRNAs in *Arabidopsis* Proc Natl Acad Sci U S A 2005 102 10 3691 3696 10.1073/pnas.0405570102 15738428 PMC553294

[B5] Hoffer P Ivashuta S Pontes O Vitins A Pikaard C Mroczka A Wagner N Voelker T Posttranscriptional gene silencing in nuclei Proc Natl Acad Sci 2011 108 1 409 414 10.1073/pnas.1009805108 21173264 PMC3017132

[B6] Lutz K Wang W Zdepski A Michael T Isolation and analysis of high quality nuclear DNA with reduced organellar DNA for plant genome sequencing and resequencing BMC Biotechnology 2011 11 1 54 10.1186/1472-6750-11-54 21599914 PMC3131251

[B7] Galbraith D Harkins K Maddox J Ayres N Sharma D Firoozabady E Rapid flow cytometric analysis of the cell cycle in intact plant tissues Science 1983 220 4601 1049 1051 10.1126/science.220.4601.1049 17754551

[B8] Arumuganathan K Earle E Estimation of nuclear DNA content of plants by flow cytometry Plant Mol Biol Rep 1991 9 3 229 241 10.1007/BF02672073

[B9] Doleźel J Greilhuber J Suda J Estimation of nuclear DNA content in plants using flow cytometry Nat Protoc 2007 2 2233 2244 10.1038/nprot.2007.310 17853881

[B10] Cires E Cuesta C Fernández Casado MÁ Nava HS Vázquez VM Fernández Prieto JA solation of plant nuclei suitable for flow cytometry from species with extremely mucilaginous compounds: an example in the genus *Viola* L. (Violaceae) An Jar Bot Madr 2011 68 2 139 154 10.3989/ajbm.2273

[B11] Loureiro J Rodriguez E Doležel J Santos C Comparison of four nuclear isolation buffers for plant DNA flow cytometry Ann Bot 2006 98 3 679 689 10.1093/aob/mcl141 16820407 PMC2803574

[B12] Bae MS Cho EJ Choi E Park OK Analysis of the *Arabidopsis* nuclear proteome and its response to cold stress Plant J 2003 36 5 652 663 10.1046/j.1365-313X.2003.01907.x 14617066

[B13] Jones AME MacLean D Studholme DJ Serna-Sanz A Andreasson E Rathjen JP Peck SC Phosphoproteomic analysis of nuclei-enriched fractions from *Arabidopsis thaliana* J Proteomics 2009 72 3 439 451 10.1016/j.jprot.2009.02.004 19245862

[B14] Khan MMK Komatsu S Rice proteomics: recent developments and analysis of nuclear proteins Phytochemistry 2004 65 12 1671 1681 10.1016/j.phytochem.2004.04.012 15276429

[B15] Repetto O Rogniaux H Firnhaber C Zuber H Küster H Larré C Thompson R Gallardo K Exploring the nuclear proteome of *Medicago truncatula* at the switch towards seed filling Plant J 2008 56 3 398 410 10.1111/j.1365-313X.2008.03610.x 18643982

[B16] Pandey A Choudhary MK Bhushan D Chattopadhyay A Chakraborty S Datta A Chakraborty N The nuclear proteome of chickpea (*Cicer arietinum* L.) reveals predicted and unexpected proteins J Proteome Res 2006 5 12 3301 3311 10.1021/pr060147a 17137331

[B17] Pendle AF Clark GP Boon R Lewandowska D Lam YW Andersen J Mann M Lamond AI Brown JWS Shaw PJ Proteomic analysis of the *Arabidopsis* nucleolus suggests novel nucleolar functions Mol Biol Cell 2005 16 1 260 269 15496452 10.1091/mbc.E04-09-0791 PMC539170

[B18] Calikowski TT Meulia T Meier I A proteomic study of the *Arabidopsis* nuclear matrix J Cell Biochem 2003 90 2 361 378 10.1002/jcb.10624 14505352

[B19] Tan F Li G Chitteti BR Peng Z Proteome and phosphoproteome analysis of chromatin associated proteins in rice (*Oryza sativa*) Proteomics 2007 7 24 4511 4527 10.1002/pmic.200700580 18022940

[B20] Aki T Yanagisawa S Application of rice nuclear proteome analysis to the identification of evolutionarily conserved and glucose-responsive nuclear proteins J Proteome Res 2009 8 8 3912 3924 10.1021/pr900187e 19621931

[B21] Pandey A Chakraborty S Datta A Chakraborty N Proteomics approach to identify dehydration responsive nuclear proteins from chickpea (*Cicer arietinum* L.) Mol Cell Proteomics 2008 7 1 88 107 17921517 10.1074/mcp.M700314-MCP200

[B22] Choudhary MK Basu D Datta A Chakraborty N Chakraborty S Dehydration-responsive nuclear proteome of rice (*Oryza sativa* L.) illustrates protein network, novel regulators of cellular adaptation, and evolutionary perspective Mol Cell Proteomics 2009 8 7 1579 1598 10.1074/mcp.M800601-MCP200 19321431 PMC2709188

[B23] Ohyama K Lawrence EP Horn D A rapid, simple method for nuclei isolation from plant protoplasts Plant Physiol 1977 60 2 179 181 10.1104/pp.60.2.179 16660054 PMC542574

[B24] Saxena PK Fowke LC King J An efficient procedure for isolation of nuclei from plant protoplasts Protoplasma 1985 128 2–3 184 189

[B25] Masuda K Takahashi S Nomura K Inoue M A simple procedure for the isolation of pure nuclei from carrot embryos in synchronized cultures Plant Cell Rep 1991 10 6–7 329 333 24221667 10.1007/BF00193152

[B26] Yamaguchi J Lim P Akazawa T Isolation and characterization of nuclei from rice embryo Cell Struct Funct 1992 17 87 92 10.1247/csf.17.87 1376640

[B27] Renouard S Cyrielle C Lopez T Lamblin F Laine E Hano C Isolation of nuclear proteins from flax (*Linum usitatissimum* L.) seed coats for gene expression regulation studies BMC Res Notes 2012 5 1 15 10.1186/1756-0500-5-15 22230709 PMC3285032

[B28] Willmitzer L Wagner KG The isolation of nuclei from tissue-cultured plant cells Exp Cell Res 1981 135 1 69 77 10.1016/0014-4827(81)90300-1 6269864

[B29] Ribeiro Silva TC Santiago Abreu I Carvalho CR Improved and reproducible flow cytometry methodology for nuclei isolation from single root meristem J Bot 2010 2010 1 7

[B30] Zhang H Zhao X Ding X Paterson AH Wing RA Preparation of megabase-size DNA from plant nuclei Plant J 1995 7 1 175 184 10.1046/j.1365-313X.1995.07010175.x

[B31] Cushman JC Galbraith DW, Bourque DP, Bohnert HJ Isolation of nuclei suitable for in vitro transcriptional studies Methods in Cell Biology. Volume 50 1995 San Diego: Academic Press 113 128 10.1016/s0091-679x(08)61026-2 8531788

[B32] Loureiro J Rodriguez E Doležel J Santos C Two new nuclear isolation buffers for plant DNA flow cytometry: a test with 37 species Ann Bot 2007 100 4 875 888 10.1093/aob/mcm152 17684025 PMC2749623

[B33] Guilfoyle TJ Galbraith DW, Bourque DP, Bohnert HJ **Isolation and characterization of plant nuclei** Methods in Cell Biology. Volume 50 1995 San Diego: Academic Press 101 112 10.1016/s0091-679x(08)61025-0 8531787

[B34] McKeown P Pendle AF Shaw PJ Hancock R Preparation of *Arabidopsis* nuclei and nucleoli The Nucleus. Volume 1 2008 New York: Humana Press 67 75 10.1007/978-1-59745-406-3_5 18951161

[B35] Carrier G Santoni S Rodier-Goud M Canaguier A Kochko Ad, Dubreuil-Tranchant C, This P, Boursiquot J, Cunff LL: **an efficient and rapid protocol for plant nuclear DNA preparation suitable for next generation sequencing methods** Am J Bot 2011 98 1 e13 e15 10.3732/ajb.1000371 21613076

[B36] Deal RB Henikoff S The INTACT method for cell type–specific gene expression and chromatin profiling in *Arabidopsis thaliana* Nat Protoc 2011 6 56 68 21212783 10.1038/nprot.2010.175 PMC7219316

[B37] Abdalla KO Thomson JA Rafudeen MS Protocols for nuclei isolation and nuclear protein extraction from the resurrection plant *Xerophyta viscosa* for proteomic studies Anal Biochem 2009 384 2 365 367 10.1016/j.ab.2008.09.049 18938124

[B38] Valkonen J Slack S Plaisted R Watanabe K Extreme resistance is epistatic to hypersensitive resistance to potato virus Y in a Solanum tuberosum ssp. andigena -derived potato genotype. Plant Dis 1994 78 12 1177 1180 10.1094/PD-78-1177

[B39] Meier U **Growth stages of mono- and dicotyledonous plants** BBCH Monograph. 2nd edition 2001: Federal Biological Research Centre for Agriculture and Forestry

[B40] Busk PK Pagés M Microextraction of nuclear proteins from single maize embryos Plant Mol Biol Rep 1997 15 4 371 376.40 10.1023/A:1007428802474

[B41] Vlasák J Effect of different disintegration techniques and media on yield and appearance of isolated nuclei Biol Plant 1981 23 6 406 413 10.1007/BF02880585

[B42] Watson JC Thompson WF Weissbach A, Weissbach H **Purification and restriction endonuclease analysis of plant nuclear DNA** Methods in Enzymology. Volume 118 1986 San Diego: Academic Press 57 75

[B43] Graham J Rickwood D Subcellular fractionation: a practical approach 1997 Oxford: IRL Press

[B44] Harford JB Bonifacino JS Subcellular fractionation and isolation of organelles Curr Protoc Cell Biol 2010 48 3.0.1 3.0.8

[B45] Millar AH Liddell A Leaver CJ Pon LA, Schon EA **Isolation and subfractionation of mitochondria from plant** Methods in Cell Biology. Volume 80 2001 San Diego: Academic Press 65 90

